# EDUCATION AND MIDLIFE COGNITIVE FUNCTIONING: EVIDENCE ABOUT MEDIATORS FROM THE HIGH SCHOOL AND BEYOND COHORT

**DOI:** 10.1093/geroni/igae098.2871

**Published:** 2024-12-31

**Authors:** John Warren, Chandra Muller, Eric Grodsky, Adam Brickman, Jennifer Manly

**Affiliations:** University of Minnesota, Minneapolis, Minnesota, United States; The University of Texas Austin, Austin, Texas, United States; University of Wisconsin Madison, Madison, Wisconsin, United States; Columbia University, New York, New York, United States; Columbia University, New York, New York, United States

## Abstract

Educational attainment is associated with cognitive functioning and risk of Alzheimer’s disease and related dementias (ADRD). However, (1) aspects of schooling that precede degree completion have rarely been considered and (2) the social and biological pathways through which education shapes ADRD risk are not well understood. Do schools’ social and academic contexts, students’ schooling outcomes, and students’ degree completion independently predict cognitive outcomes? If so, how do life course social and biological factors mediate the effects of various dimensions of education? This study included a nationally representative sample of 12,530 Americans followed prospectively from high school through age ~60. We estimated associations between aspects of schooling and multiple dimensions of cognition, before and after adjustment for confounders. We then considered the mediating roles of midlife socioeconomic factors (e.g., income, financial precarity, debt, bankruptcy risk) and biological/health factors (e.g., cardiovascular disease, immunological function, inflammatory markers). We employ a variety of methodological approaches informed by recent advances in approaches to mediation analysis within causal frameworks. Education is more than just degree attainment. Understanding how and through what pathways education shapes cognitive functioning and ADRD risk requires longitudinal data with nuanced information about schools, schooling, and student performance.

